# Relationship between thyroid hormone and sex hormone levels and non-suicidal self-injury in male adolescents with depression

**DOI:** 10.3389/fpsyt.2022.1071563

**Published:** 2022-12-21

**Authors:** Jiahui Ma, Mingming Zhao, Gengyun Niu, Zhifei Wang, Shan Jiang, Zengxun Liu

**Affiliations:** ^1^Department of Psychiatry, Jining Medical University, Jining, China; ^2^Department of Psychiatry, Shandong Mental Health Center, Jinan, China

**Keywords:** non-suicidal self-injury, thyroid hormone, sex hormones, adolescent, depression

## Abstract

**Objective:**

Non-suicidal self-injury (NSSI) is the intentional and repeated direct injury to one’s bodily tissues or organs without the intent to die, which is not socially sanctioned and does not result in death. This study will be the first to explore the relationship between NSSI behavior and thyroid hormone and sex hormone levels in male adolescents with depression.

**Methods:**

Among the inpatients in the children’s ward of Shandong Mental Health Center, eighty male patients with first-episode depressive disorder were randomly selected. Forty male adolescent depressed patients with NSSI behaviors were set as the NSSI group, and forty male adolescent depressed patients without NSSI behaviors were set as the No-NSSI group. Their thyroid hormones (free triiodothyronine, free thyroxine, and thyroid stimulating hormone) and sex hormones (estradiol, progesterone, and testosterone) were measured, and the severity of self-injury in the NSSI group was assessed using the Adolescent Self-Injury Questionnaire. The NSSI group was tested again after 6 weeks of sertraline treatment for biological indicators and assessed by the Self-Injury Questionnaire to compare the hormonal differences between the NSSI group and the No-NSSI group and compare the differences of each index before and after treatment in the NSSI group.

**Results:**

T3/T4 (*p* = 0.001) and FT3 (*p* = 0.023), TSH levels (*p* < 0.001) were lower in the NSSI group than in the No-NSSI group before treatment, and FT4 (*p* = 0.036) and T (*p* < 0.001) levels were higher than in the No-NSSI group. T3/T4 levels were higher in the NSSI group after treatment (*p* < 0.001). FT4 (*p* < 0.001) and T (*p* = 0.001) levels and self-injury questionnaire scores (*p* < 0.001) decreased after treatment in the NSSI group. In the NSSI group at baseline, FT4 levels were negatively correlated with self-injury questionnaire scores (*r* = −0.459, *p* = 0.003) and testosterone levels were positively correlated with self-injury questionnaire scores (*r* = 0.383, *p* = 0.015), and in the NSSI group after treatment, FT4 difference was negatively correlated with self-injury questionnaire score reduction rate (*r* = −0.037, *p* = 0.019), and testosterone difference was positively correlated with self-injury questionnaire score reduction rate (*r* = 0.424, *p* = 0.006). Logistic regression analysis showed that low TSH and high testosterone levels were independent risk factors for the development of non-suicidal self-harming behaviors in male adolescent depressed patients.

**Conclusion:**

Changes in thyroid hormone and sex hormone levels may be associated with non-suicidal self-injurious behavior in male adolescent depressed patients.

## Introduction

Non-suicidal self-injury (NSSI) is the intentional and repeated direct injury to one’s bodily tissues or organs without the intent to die, which is not socially sanctioned and does not result in death (1). In recent years, NSSI behavior has received increasing attention across the world. It has been noted that NSSI behaviors tend to occur in adolescence. A 2014 meta-analysis showed that the incidence of self-injury was 17.2% in adolescents, 13.4% in young adults, and 5.5% in adults (2) and that adolescents who began self-injury at or before age 12 experienced more lifetime self-injurious behaviors than those who began self-injury after age 17 (3). Depressive disorder is a mood disorder with depressed mood, loss of interest, and diminished volitional activity as core symptoms, and some studies have proposed that adolescents with depression exhibit more and more consistent self-injurious behavior than those without depression (4). In the same way that depressive disorders are a risk factor for the development of NSSI behaviors, NSSI behaviors can be used to predict the development of depressive disorders and suicidal behaviors, and there is substantial evidence that NSSI is a better predictor of suicidal behavior than a history of suicide attempts (5), and suicidal ideation increases with the increasing frequency and severity of NSSI behaviors (6).

Self-injury is a complex behavior that is the result of the intersection of psychosocial (7), behavioral (8), and neurobiological mechanisms. The relationship between NSSI behavior and the endocrine system is unclear, and studies have confirmed that non-suicidal self-injury is associated with dysregulation of the hypothalamic-pituitary-adrenal axis (9, 10). It has also been proposed that the endogenous opioid system mediates the emotional regulation of non-suicidal self-injurious behavior through its involvement in the regulation of pain and emotion (11–13). Increasingly, attention is being focused on the relationship between thyroid and gonadal hormones and NSSI behavior. Duval (14) suggested that lack of TSH response to thyrotropin releasing hormone (TRH) may be a risk factor for suicide, and one study found that serum FT4 levels were lower in depressed suicide attempters than in non-suicide attempters, but there was no significant difference in TSH and FT3 levels (15). Some medications have also been found to be effective in relieving a range of symptoms, including but not limited to self-injury, in adolescents with gender dysphoria by shutting down the hypothalamic pituitary gonadal axis (HPG), resulting in the suspension of testosterone or estrogen production (16).

Considering that the relationship between NSSI behavior and thyroid hormones and sex hormones is poorly understood, we conducted this study to investigate the possibility of a relationship between NSSI behavior and the hypothalamic-pituitary-thyroid (HPT) axis and the hypothalamic-pituitary-gonadal (HPG) axis.

## Patients and methods

### Study design and participants

This study randomly selected 80 male patients with first-episode depressive disorder who were hospitalized in the children’s ward of Shandong Mental Health Center from October 1, 2021 to January 31, 2022. The inclusion criteria were (1) aged between 12–18 years and never having received any medication or psychosocial interventions prior to this time, and (2) meeting the diagnostic criteria for persistent depressive disorder in the Diagnostic and Statistical Manual of Mental Disorders (DSM-5). The exclusion criteria were as follows: (1) current or previous history of other psychiatric disorders; (2) comorbid organic brain disorders, traumatic brain injury, etc.; (3) disorders affecting thyroid function and testicular function (e.g., primary hypothyroidism, orchitis); (4) previous history of suicide attempts (self-injurious behavior with the intent to end life); and (5) current or previous history of substance dependence or abuse. According to the diagnostic criteria of non-suicidal self-injury in DSM-5, 40 male adolescent depressed patients with NSSI behaviors were set as the NSSI group, and 40 male adolescent depressed patients without NSSI behaviors were set as the No-NSSI group.

This study was conducted in accordance with the Declaration of Helsinki. The study protocol was approved by the Ethics Committee of Shandong Mental Health Center (2021-R102). All patients and family members participating in the study were informed and voluntarily signed the informed consent form.

### Assessment scales

Ottawa self-injury inventory (OSI): The OSI is a self-rating scale containing 28 items to rate the frequency, method, and motivation of self-injury in the past year and addresses self-injurious behavior in response to negative emotions, stressful events, and self-injury addiction (17). This scale has been studied as a valid and reliable assessment tool for adolescents (18), and the Chinese version has good measurement validity (19).

The Modified version of Adolescents Self-Harm Scale: The development of this questionnaire was based on the Functional Assessment of Self- Mutilation (FASM) by Lloyd et al. (20) and later revised by Feng et al. (21). It contains 19 types of self-injury and consists of two parts: the number of self-injuries (4 levels) and the degree of harm to the body (5 levels). The score = the number of occurrences × the degree of severity. A score ≥ 1 is considered to indicate self-injurious behavior, and a higher score indicates more serious NSSI behavior. This scale has a good measurement index with an internal consistency coefficient of 0.85.

Hamilton Depression Scale-17 item version (22) (HAMD-17): commonly used to assess the degree of clinical depression, mainly including cognitive impairment, sleep disturbance, somatization symptoms, and other dimensions, with higher scores indicating more severe depression; some studies have shown good internal consistency (23).

### Research methodology

A questionnaire was administered to the patients on the first day after admission. Fasting venous blood was collected in the early morning of the next day, and patients were asked to fast and abstain from food and water after 12:00 a.m. on the day before blood collection. All peripheral blood samples were collected and centrifuged for 10,000 rmp/min for 10 min at 4°C, and then all blood samples were tested for sex hormones and thyroid hormones using the i2000SR fully automated chemiluminescence immunoanalyzer developed by Abbott, USA. All samples were analyzed by the same analyst, who was blind to the sample sources. The adolescent self-harm questionnaire was retested after 6 weeks, and the subtraction rate was calculated as (pre-treatment score–post-treatment score)/pre-treatment score. All biological indicators were reviewed.

### Endocrinological assays

Thyroid function was assessed according to free triiodothyronine (FT3), free thyroxine (FT4), thyrotropin (TSH), and FT3/FT4 ratio. Gonadal function was assessed according to estradiol (E2), progesterone (Prog), and testosterone (T). The reference ranges were 1.80–4.20 pg/ml for FT3, 0.87–1.85 ng/dl for FT4, and 0.50–7.30 μIU/ml for TSH. The reference ranges were 0–84 pg/ml for E2, 0.10–0.84 ng/ml for Prog, and 6.27–27.98 nmol/L for T.

### Treatment method

The current use of antidepressants in children and adolescents is dominated by selective serotonin reuptake inhibitors (SSRIs), and sertraline is the most commonly used (24). Clinical studies have shown that sertraline is significantly more effective than placebo in the treatment of depression in children and adolescents (25), and has few and mild adverse effects that are well tolerated by most children (26). Most antipsychotic drugs affect thyroid hormones, however, it has been shown that sertraline use has no effect on thyroid hormones (27, 28). Therefore, the NSSI group was treated with sertraline monotherapy with a starting dose of 50 mg/d, which was gradually increased to 100–200 mg/d within 2 weeks for 6 weeks, and no other antidepressants, antipsychotics or mood stabilizers were applied during the treatment period, and lorazepam could be given intermittently orally at night if the patients developed severe insomnia disorder.

### Statistical methods

SPSS 26.0 was used for data analysis, and continuous data with a normal distribution are expressed as the mean ± standard deviation (±s) with a *t*-test, while continuous data with a non-normal distribution are expressed as the median (interquartile range, IQR) with a non-parametric test. Differences between the two groups were tested by independent sample *t*-test, and within-group changes before and after treatment were analyzed by paired sample *t*-test; the correlation between hormone levels and the severity of self-injury was tested by Spearman correlation analysis. The ratios that differed between groups were included in a binary logistic regression equation to analyze and explore the relationship between self-injury and hormones. Receiver operating characteristic (ROC) curves were used to analyze the value of each hormone in assessing self-injurious behavior.

## Results

### Demographics

The mean age of the NSSI group was 15.00 ± 2 and the mean years of education was 7.78 ± 2.213, while the mean age of the No-NSSI group was 15.50 ± 2 and the mean years of education was 7.98 ± 1.132, with no significant differences when compared (*P* > 0.05).

### Comparison of the indexes at baseline between the experimental and No-NSSI groups

As shown in Table 1, the level of FT3 was lower in the NSSI group than in the No-NSSI group (*p* = 0.023), the level of FT4 was higher in the NSSI group than in the No-NSSI group (*p* = 0.036), T3/T4 in the NSSI group was lower than that in the No-NSSI group (*p* = 0.001), and the level of TSH was lower in the NSSI group than in the No-NSSI group (*p* < 0.001). The testosterone level in the group was higher than that in the No-NSSI group (*p* < 0.001), and the HAMD-17 score in the NSSI group was higher than that in the No-NSSI group (*p* = 0.003).

**TABLE 1:** Comparison of indicators at baseline between the two groups.

Indicator	NSSI group	No-NSSI group	*t*	*p*
	***N* = 40**	***N* = 40**		
FT3 (pg/ml)	3.38 ± 0.35	3.59 ± 0.45	–2.331	**0.023**
FT4 (ng/ml)	1.13 ± 0.24	1.03 ± 0.22	2.098	**0.036**
T3/T4	2.92 ± 1.00	3.47 ± 0.69	–3.329	**0.001**
TSH (μIU/ml)	2.78 ± 1.34	3.86 ± 1.22	–3.773	**< 0.001**
E2 (pg/ml)	30.32 ± 11.10	28.20 ± 10.28	0.087	0.931
Prog (ng/ml)	0.46 ± 0.33	0.55 ± 0.34	–1.670	0.095
T (nmol/L)	21.96 ± 6.43	16.82 ± 5.68	3.788	**< 0.001**
HAMD-17	25.28 ± 5.63	20.85 ± 7.27	3.044	**0.003**

FT3, free triiodothyronine; FT4, free thyroxine; TSH, thyroid stimulating hormone; E2, estradiol; Prog, progesterone; T, testosterone; HAMD-17, Hamilton Depression Scale-17 item version. Bold value means *P* < 0.05.

### Comparison of the indexes before and after treatment in the NSSI group

As shown in Table 2, after the treatment in the NSSI group, the level of FT4 was decreased (*p* < 0.001), T3/T4 was higher (*p* < 0.001), the level of testosterone was decreased (*p* = 0.001), and the score of the self-injury questionnaire was decreased (*p* < 0.001).

**TABLE 2:** Comparison of the indexes before and after treatment in the non-suicidal self-injury (NSSI) group.

Indicator	Before treatment	After treatment	*t*	*p*
FT3 (pg/ml)	3.38 ± 0.35	3.26 ± 0.38	1.874	0.068
FT4 (ng/ml)	1.13 ± 0.24	0.90 ± 0.30	5.800	**< 0.001**
T3/T4	2.92 ± 1.00	3.46 ± 2.17	4.086	**< 0.001**
TSH (μIU/ml)	2.78 ± 1.34	3.09 ± 1.57	1.727	0.084
E2 (pg/ml)	30.32 ± 11.10	33.15 ± 13.04	–1.746	0.089
Prog (ng/ml)	0.46 ± 0.33	0.48 ± 0.27	–1.766	0.077
T (nmol/L)	21.96 ± 6.43	18.86 ± 7.16	3.767	**0.001**
Self-injury questionnaire score	29.80 ± 11.00	7.95 ± 5.07	15.785	**< 0.001**

FT3, free triiodothyronine; FT4, free thyroxine; TSH, thyroid stimulating hormone; E2, estradiol; Prog, progesterone; T, testosterone. Bold value means *P* < 0.05.

### Correlation between hormone levels and self-injury questionnaire scores before and after treatment in the NSSI group

**FIGURE 1:**
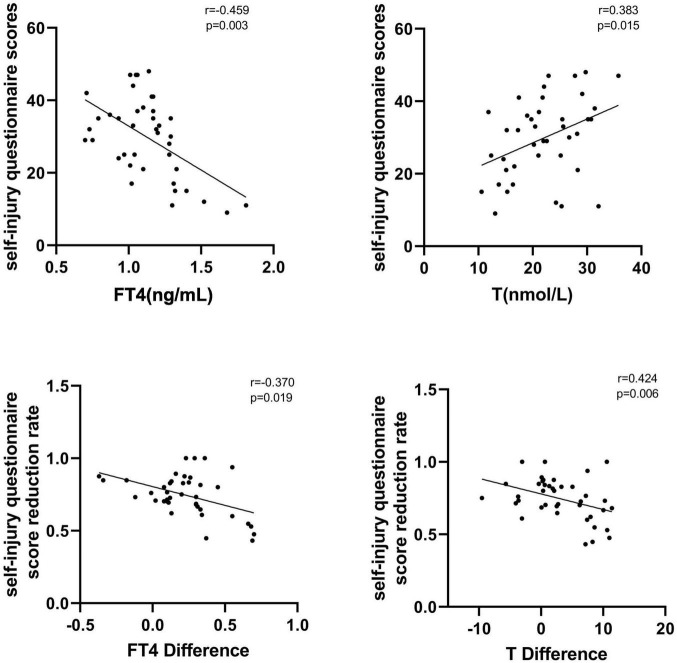
Correlation between hormone levels and self-injury questionnaire scores.

In the NSSI group, FT4 levels were negatively correlated with self-injury questionnaire scores at baseline (*r* = −0.459, *p* = 0.003), testosterone levels were positively correlated with self-injury questionnaire scores (*r* = 0.383, *p* = 0.015), and FT3, TSH, E2, and Prog levels were not significantly correlated with self-injury questionnaire scores (*P* > 0.05). After treatment in the NSSI group, FT4 difference was negatively correlated with self-injury questionnaire score reduction rate (*r* = −0.370, *p* = 0.019), while testosterone difference was positively correlated with self-injury questionnaire score reduction rate (*r* = 0.424, *p* = 0.006), and FT3, TSH, E2, and Prog differentials were not significantly correlated with self-injury questionnaire score reduction rate (*P* > 0.05). See Figure 1 for details.

### Logistic regression analysis of factors influencing NSSI behavior in male adolescents with depression

**FIGURE 2:**
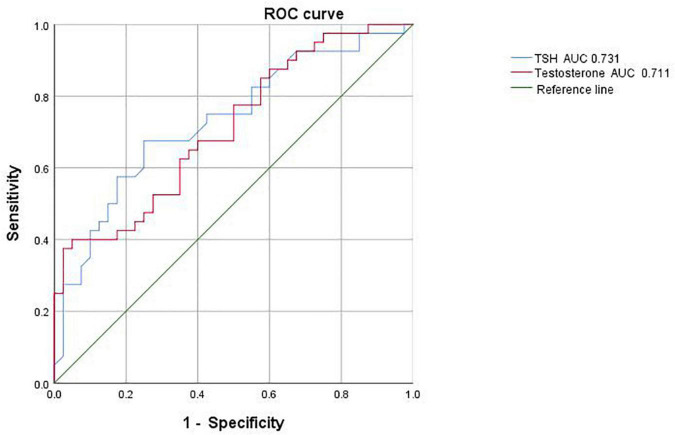
Receiver operating characteristic (ROC) curve analysis of male adolescent depressed patients with non-suicidal self-injury (NSSI).

Receiver operating characteristic curve analysis based on the above indicators showed that the critical value for TSH prediction was 3.225 μIU/ml, with an AUC of 0.731, sensitivity of 67.5% and specificity of 75%; the critical value for testosterone prediction in male adolescent depressed patients with NSSI behavior was 23.995 nmol/L, with an AUC of 0.711, sensitivity of 40% and specificity of 95% (Figure 2).

## Discussion

In this paper, we investigated the relationship between thyroid hormones, sex hormones, and non-suicidal self-injurious behavior in male adolescent depressed patients and explored for the first time the correlation between thyroid hormones, sex hormones and the severity of non-suicidal self-injurious behavior. We found that male adolescent depressed patients with non-suicidal self-injurious behavior had significantly lower FT3 and TSH levels and significantly higher FT4 and testosterone levels than male adolescent depressed patients without self-injurious behavior.

In recent years, non-suicidal self-injurious behaviors have become increasingly frequent in the adolescent population and have become a significant cause of mental health problems in adolescents. There is a growing interest in the mechanisms underlying the occurrence of this behavior, which remains inconclusive. NSSI is a complex behavior that emerges through the intersecting effects of social, psychological, and biological mechanisms. Although social and psychological contributions to the development of NSSI risk are relatively well established and have guided the development of effective psychosocial treatments for self-harm, the biological mechanisms behind NSSI are just beginning to emerge (29), and current research on the relationship between NSSI behavior and neurobiological factors remains focused on the opioid system (11–13) and the hypothalamic–pituitary–adrenal (HPA) axis (9, 10).

Thyroid hormones are important in the development of the mammalian brain and play a role in the migration and differentiation of nerve cells, synaptogenesis, and myelination (30). An increasing number of people are now aware of the link between thyroid function and depression (31, 32). It has been proposed that serum T3 and FT3 are significantly lower in patients with first-episode depression before treatment (33), that there is a significant correlation between FT4 levels and depression severity, and that higher FT4 concentrations are significantly associated with more severe depression (34). It has also been demonstrated that lower TSH and higher T4 levels are associated with depressive syndromes in men, but only higher T4 levels are associated with depressive syndromes in women (35). This change (decrease in T3, FT3, TSH, and increase in FT4) is associated with depression, probably because the bioactive effect of thyroid hormones on mental activity acts mainly on the metabolism of central neurotransmitters. That is, thyroid hormones can promote elevated mood by accelerating the rate of synthesis and metabolism of central neurotransmitters (36). If the levels of T3, FT3, and TSH decrease, the synthesis and metabolism of central neurotransmitters will slow down accordingly, resulting in depressive symptoms such as depressed mood. It has been suggested that thyroid hormones may be promising biomarkers of suicide risk in patients with depression (37). It has also been shown that inter-individual differences in serum thyroid hormone levels correlate significantly with specific aspects of personality, so thyroid hormone disorders can lead to unstable personality traits in individuals, which may consequently lead to impulsivity and negative behavior (38). However, the relationship between thyroid hormones and self-injurious behavior in depressed patients is still being studied.

The results of this study showed that male adolescent depressed patients with non-suicidal self-injurious behavior had higher FT4 levels than the No-NSSI group, and the NSSI group had significantly lower FT4 levels after a period of treatment. Studies on the relationship between non-suicidal self-injurious behavior and thyroid function at this stage are still in the exploratory stage, and the conclusions reached are inconsistent. Flach et al. (39) found that female patients with non-suicidal self-injurious behavior had lower FT3 levels and higher FT4 levels *in vivo* than the healthy population, and that the FT3/FT4 ratio was negatively correlated with the degree of depression. This is consistent with the conclusions reached in the present study. Meanwhile, the present study also demonstrated that T3/T4 was significantly lower in self-injured patients than in depressed patients without self-injurious behavior, and that T3/T4 levels increased after improvement in self-injurious behavior, thus indicating that self-injurious behavior can lead to a decrease in T4 to T3 conversion. This may be due to an interruption in the conversion of hormones from T4 deiodine to T3 in self-injured patients, resulting in lower FT3 levels. Abnormal T4 to T3 conversion can lead to symptoms such as fatigue, depression, and difficulty concentrating, suggesting that these symptoms should be taken into account when self-injured patients are seen.

Jose et al. (40) showed that suicidal ideation in schizophrenic patients may be associated with elevated FT4 levels, and we can speculate that there is some commonality in the biological mechanisms of suicidal and self-injurious behaviors (41). Our study also concluded that depression in male adolescents with non-suicidal self-injury behavior decreased after a period of treatment on the Self-Injury Scale, and the rate of reduction was negatively correlated with the FT4 difference. It has been suggested (42) that cognitive function in depressed patients is negatively correlated with T4 levels. Existing studies have demonstrated that impairment of cognitive function can lead to the development of NSSI behaviors (43, 44). Existing studies have found that non-suicidal self-injury populations exhibit functional abnormalities in some brain regions, such as the orbitofrontal, prefrontal, amygdala, cingulate, hippocampus, and corpus callosum (45, 46), which are core brain regions for emotional processing and cognition. We can therefore hypothesize that elevated FT4 levels lead to impaired cognitive function, which further induces patients to adopt self-injurious behaviors in response to negative emotions.

Peng et al. (15) found that low levels of TSH predicted the occurrence of self-injurious behaviors. In our study, we also concluded that low levels of TSH can be an independent influence on non-suicidal self-injurious behavior, which may be due to the dysfunctional negative feedback regulation of the HPT axis in male adolescent depressed patients with non-suicidal self-injurious behavior, resulting in the inability to increase TSH release through negative feedback after a decrease in FT3 levels, which further affects FT3 levels.

The hypothalamic-pituitary-gonadal (HPG) axis is a neuroendocrine system that regulates the release of gonadotropin-releasing hormone, gonadotropin, and sex hormones through feedback and negative feedback between the hypothalamus, pituitary gland, and gonads. At this stage, an increasing number of researchers are focusing on the relationship between sex hormones and depression. Depression shows gender differences both in terms of prevalence and symptoms (47), and this gender difference begins to appear during adolescence, which is a critical stage in the development of secondary sexual characteristics in adolescents, so it can be hypothesized that the occurrence of mood disorders in adolescents is mostly associated with the hypothalamic-pituitary-gonadal axis. Studies have shown that both testosterone and estradiol exhibit anxiolytic and antidepressant-like effects in gonadectomized male rats (48). It has been shown that sex hormone replacement therapy can help improve depressive symptoms (49). There is growing evidence that sex hormones fluctuate in people with depression, which can affect mood and cognitive function (50).

This study found that testosterone levels were significantly higher in male adolescent depressed patients with NSSI behaviors than in male adolescent depressed patients without NSSI behaviors, and the severity of self-injurious behaviors was positively correlated with testosterone levels. A recent analysis of data showed that long-term testosterone use leads to emotional instability and that testosterone use is independently associated with intentional self-harming behavior (51), a finding that is consistent with our findings.

At this stage, there are more studies on testosterone levels and suicidal behavior, and in 2012, Sher et al. (52) suggested that in patients with bipolar disorder, testosterone levels were positively associated with the number of suicide attempts in male patients, and in 2013, Sher again hypothesized that high testosterone levels are associated with aggression and suicidal behavior in adolescents and that low testosterone levels are associated with suicidal behavior in older adults (53). This may be due to testosterone-mediated attenuation of prefrontal cortex (PFC) function, altered PFC-limbic functional connectivity, and overactivation of limbic areas, which impairs the emotion regulation system and thus increases the risk of suicide in adolescents (54). On the other hand, it has been demonstrated that genetic variation in the efflux transport protein permeability glycoprotein (P-gp) is associated with self-injury and suicide attempts (55–57), and a recent study showed that testosterone is an endogenous substrate for P-gp, suggesting that intra-cerebral transport of androgens such as testosterone may be regulated by P-gp (58). Thus, impaired P-gp function, or the use of competing P-gp substrates, may alter androgen transport, leading to elevated androgen levels in the brain, a finding that also provides a physiological basis for the association between elevated brain testosterone levels and self-injurious and suicidal behavior. A survey conducted in Shandong, China, in 2015 also showed that male suicide attempters had significantly higher testosterone levels than their community peers (59). Although these studies did not use NSSI behaviors as a starting point, as we mentioned before, suicide and NSSI behaviors have some commonalities in biological mechanisms, so it can still be confirmed that changes in testosterone levels can influence the occurrence of NSSI behaviors to some extent.

We showed that TSH and testosterone levels can predict the occurrence of NSSI behaviors by analyzing ROC curves. In this study, we found that the best cutoff values for TSH and testosterone to predict NSSI behavior were 3.225 μIU/ml and 23.995 nmol/L, respectively. No studies have reported cutoff values for hormone levels to infer self-injurious behavior, and our findings could provide more sensitive biological markers for clinical purposes.

There are some shortcomings in this study. First, our sample size was relatively small, and the observation time was short; second, we did not collect healthy people as controls, which may have some influence on the study results; again, the scales we used were mostly self-assessment scales, and recall bias may occur when answering, which may also have some influence on the accuracy of the results.

## Conclusion

This study suggests that changes in thyroid hormone and sex hormone levels may be associated with non-suicidal self-injurious behavior in male adolescents with depression. This study found that male adolescent depressed patients with NSSI behaviors had higher FT4 and testosterone levels and low FT3 and TSH levels compared to male adolescent depressed patients without NSSI behaviors. The severity of self-injury was negatively correlated with FT4 levels and positively correlated with testosterone levels. And, low TSH and high testosterone levels were independent risk factors for the development of non-suicidal self-injurious behavior in male adolescent depressed patients. In summary, changes in thyroid and gonadal hormone levels should be closely monitored when psychiatrists treat NSSI behaviors.

## Data availability statement

The original contributions presented in this study are included in the article/supplementary material, further inquiries can be directed to the corresponding author.

## Ethics statement

The studies involving human participants were reviewed and approved by Shandong Mental Health Center. Written informed consent to participate in this study was provided by the participants’ legal guardian/next of kin.

## Author contributions

JM participated in the design of the study, analyzed the data, and wrote this manuscript. MZ, GN, ZW, and SJ did the online survey, data collection, and logic checking. ZL revised the manuscript. All authors reviewed and approved the manuscript.
